# The Role of Polyphenols in Regulating Skeletal Muscle Development and Homeostasis: Molecular Mechanisms and Potential Medical Applications

**DOI:** 10.1002/mnfr.70476

**Published:** 2026-04-25

**Authors:** Roberto Mattioli, Daniel Di Risola, Carmen Chiaradia, Ivan Dimauro, Luciana Mosca, Guglielmo Duranti, Roberta Ceci

**Affiliations:** ^1^ Department of Biochemical Sciences “A. Rossi Fanelli” Sapienza University of Rome Rome Italy; ^2^ Laboratory of Biochemistry and Molecular Biology Department of Movement Human and Health Sciences Università degli Studi di Roma “Foro Italico” Rome Italy; ^3^ Laboratory of Biology and Human Genetics Department of Movement Human and Health Sciences Università degli Studi di Roma “Foro Italico” Rome Italy; ^4^ Center for Research in Neurobiology 'Daniel Bovet' (CRiN) Sapienza University of Rome Rome Italy

**Keywords:** development, inflammation, muscle cells, oxidative stress, polyphenols

## Abstract

A healthy lifestyle, characterized by moderate physical activity, appropriate caloric intake, and a diet rich in fruits and vegetables, contributes to maintaining overall health and preventing several degenerative diseases. Within this context, the health of the muscular system also plays a pivotal role. Increasing evidence highlights the importance of a balanced diet, in combination with regular physical exercise, in preserving muscle function and integrity. Polyphenols, present in fruits, vegetables, and plant‐derived foods, have emerged as key allies in counteracting oxidative stress and inflammation, processes that affect muscle cell health. These compounds are involved in the regulation of muscle cell development and differentiation, as well as in the regeneration processes following injury or excessive physical exertion. Through their ability to modulate reactive oxygen species levels, inflammation, and specific cellular pathways, polyphenols are capable of influencing muscle development and homeostasis. This review provides a comprehensive overview of current knowledge regarding the impact of polyphenols on skeletal muscle growth, development, and maintenance, with a focus on their mechanisms of action and therapeutic potential. Recent and innovative extraction and administration strategies, aimed at overcoming some limitations that normally characterize experimentation with bioactive molecules such as polyphenols, are considered and discussed in a prospective view.

## Graphical abstract

Graphical abstract describes the impact of polyphenols on signal transduction pathways involved in the development, differentiation and regeneration of muscle tissue.

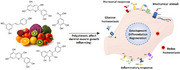

**Abbreviations**
ALS: amyotrophic lateral sclerosis;
AMPK: AMP‐activated protein kinase;
AREs: antioxidant response elements;
BMCs: bovine skeletal muscle cells;
cAMP: cyclic AMP;
CAT: catalase;
CCA: chronic contractile activity;
CK: creatine kinase;
COX: cyclooxygenases;
CPE: flavonoid‐rich cocoa extract;
DMD: Duchenne muscular dystrophy;
FAK: adhesion kinase;
FFAs: free fatty acids;
GPX: glutathione peroxidise;
GSH: glutathione;
GST: glutathione s‐transferase;
HFD: high‐fat diet;
HO‐1: heme oxygenase‐1;
IGF‐1: insulin‐like growth factor 1;
IL‐1β: interleukin‐1β;
IL‐6: interleukin‐6;
IRS: insulin receptor substrates;
KO: knockout;
LDH: lactate dehydrogenase;
LOX: lipoxygenases;
LPS: lipopolysaccharide;
MAPK: mitogen‐activated protein kinase;
Mb: myoglobin; M‐CSF,
M‐CSF: macrophage colony‐stimulating factor;
MRFs: myogenic regulatory factors;
MuSCs: muscle stem cells;
Nrf2: nuclear factor erythroid 2‐related factor 2;
PCA: protocatechuic acid;
PCOs: oligomeric proanthocyanidins;
ROS: reactive oxygen species;
RSV: resveratrolo;
SIRT1: sirtuin 1;
SOD: superoxide dismutase;
STEE: sugarcane top ethanol extract;
TNF‐α: tumor necrosis factor‐α

## 1 Introduction

Muscle health is important for maintaining a good quality of life. In addition to enabling the normal performance of daily activities, it plays a crucial role in preventing injuries, improving strength and endurance, reducing the risk of falls and osteoporosis, promoting balance and agility, facilitating fat metabolism, and regulating mood.

To preserve muscle health, it is advisable to engage in moderate but regular physical activity, avoid both excessive exertion and sedentary behavior, maintain proper posture (including through postural exercises), follow a diet rich in fruits and vegetables, and adopt an overall healthy lifestyle. Conversely, an unhealthy habit such as physical inactivity, and poorly balanced diet, not only contribute to the onset of degenerative diseases, like cardiovascular, and neurodegenerative diseases, or cancer but also definitively impair muscle health.

In fact, oxidative stress, that occurs when the production of ROS exceeds the endogenous antioxidant defences, is a key factor in compromising muscle health. Oxidative stress can lead to muscle atrophy, a degenerative condition marked by a loss of muscle mass and consequently strength [1]. However, it is important to distinguish between low‐grade oxidative stress, as occurs during regular physical exercise, and high‐grade or pathological oxidative stress. During normal physical activity, ROS are produced as part of cellular metabolism. These ROS can have beneficial effects by activating genes involved in antioxidant defences and detoxification [2]. However, when ROS levels exceed the cellular redox capacity such as during intense physical exertion, they can negate the benefits induced by exercise, causing cellular damage and dysfunctions [3].

Inflammation, another major contributor to degenerative diseases, also plays a critical role in muscle mass loss. This is observed in conditions like inflammatory myopathies and chronic obstructive pulmonary disease [4]. Pro‐inflammatory cytokines, particularly interleukin‐17 (IL‐17) and interleukin 6 (IL‐6) are key mediator of these effects. Elevated IL‐17 mRNA levels, for instance, have been detected in muscle biopsies from patients with Duchenne muscular dystrophy (DMD), suggesting a pathogenetic role for this cytokine. IL‐6 is also strongly implicated in muscle development and inflammatory response. Its expression is detectable in skeletal muscle cells as early as 30 min after the start of exercise. Furthermore, following muscle injury, IL‐6 levels temporarily increase, returning to normal in control mice within approximately 7 days, whereas in *mdx* mice, a model of DMD, elevated IL‐6 levels persist [4].

Elevated and persistent levels of IL‐6 have been linked to chronic pathological conditions such as muscle atrophy and inflammation, through the reduction of insulin‐like growth factor (IGF‐1) expression. Conversely, the absence or deficiency of IL‐6 impairs the proliferation and differentiation of myoblasts by disrupting the JAK/STAT3 signaling pathway.

Both oxidative stress and inflammation can significantly contribute to mitochondrial dysfunction primarily by damaging proteins, membrane lipids, mitochondrial DNA, as well as by disrupting normal mitochondrial processes. These impairments lead to reduced ATP production, increased susceptibility to apoptosis, and altered calcium (Ca^2+^) homeostasis. As a result, mitochondrial dysfunction is strongly implicated in the skeletal or cardiac muscle cell pathologies. Pathologies resulting from alterations in mitochondrial functions are known as mitochondriopathies. When affects the muscular system, they can lead to symptoms such as muscle weakness, cramps, exercise intolerance, and sarcopenia [5, 6, 7].

In this context, identifying molecules capable of counteracting inflammation and oxidative stress is of critical importance. A balanced diet rich in fruits and vegetables, can supply the body with bioactive molecules beneficial for the prevention and management of muscle related disorders. Fruits and vegetables, in fact, are natural sources of antioxidants and micronutrients that help mitigate oxidative stress and modulate inflammatory responses [8].

For instance, vitamin C, is essential for the synthesis of collagen, a structural protein vital for bone formation and connective tissue integrity and muscle function. Each muscle fiber is encased in a collagen scaffold that provides mechanical support, facilitate force transmission, and helps maintain the structural integrity of muscle tissue during movement. After exercise or trauma, collagen contributes to muscle regeneration by supporting the formation of new extracellular matrix, essential for tissue repair. Finally, collagen improves muscle resilience and reduces the risk of tears, strains, and injuries [9, 10, 11]. Fruits and vegetables also contain high levels of vitamin E (which protects cell membranes from damage caused by ROS), Coenzyme Q10 (a key player in the electron transport chain), magnesium (a cofactor in many energy‐producing reactions), and potassium (which is essential for muscle contraction).

In this context, a prominent role is certainly played by polyphenols. These belong to a heterogeneous and varied group of natural organic compounds present in fruits, vegetables, legumes, seeds, and whole grains. Polyphenols, such as those found in extra‐virgin olive oil, green tea, pomegranate, chocolate, wine, etc., are renowned for their powerful antioxidant and anti‐inflammatory properties. They have demonstrated a pivotal role in the prevention and management of numerous viral, microbial, metabolic, and degenerative diseases including cardiovascular diseases, neurodegenerative diseases, as well as cancer [12].

Despite the numerous studies investigating the effects of polyphenols on the physiology and pathology of skeletal muscle, the growing body of evidence remains fragmented. In particular, studies often focus on individual mechanisms, such as antioxidant activity, anti‐inflammatory properties, and mitochondrial or metabolic modulation, without providing a clearly integrated framework describing how these mechanisms interact with one another. However, oxidative stress, inflammation, and mitochondrial dysfunction are closely interconnected processes that can collectively influence skeletal muscle development, differentiation, and homeostasis.

The aim of this review is to present an updated overview on the effects of polyphenols on skeletal muscle, with particular a focus on muscle cell development, cellular homeostasis, and muscle diseases related to aging, genetic factor, and metabolic conditions.

In this review, particular effort is devoted to providing a comprehensive and as integrated as possible overview of the various processes influenced by polyphenols that ultimately affect the development and health of skeletal muscle. By integrating findings from molecular, cellular, and physiological studies, this review aims to offer a broader framework for understanding the potential clinical and nutritional applications of polyphenols, while also highlighting existing limitations and outlining future research directions in this field.

For this purpose, to identify relevant literature, a search was conducted in the PubMed database (https://pubmed.ncbi.nlm.nih.gov/) up to January 2026 using the following keywords: polyphenols, skeletal muscle, skeletal muscle cells, C2C12, development, differentiation, homeostasis, health, disease, oxidative stress, inflammation, mitochondrial dysfunction, metabolism, and metabolic disorders.

Articles focusing on cardiac muscle or smooth muscle cells were excluded from the analysis, even when these terms were not explicitly included among the search keywords.

Among the retrieved publications, studies published from 2003 onwards were considered for this review. Particular emphasis was placed on more recent literature (from 2020 onwards), which represents approximately 56% of the studies included.

## 2 Polyphenols

**FIGURE 1:**
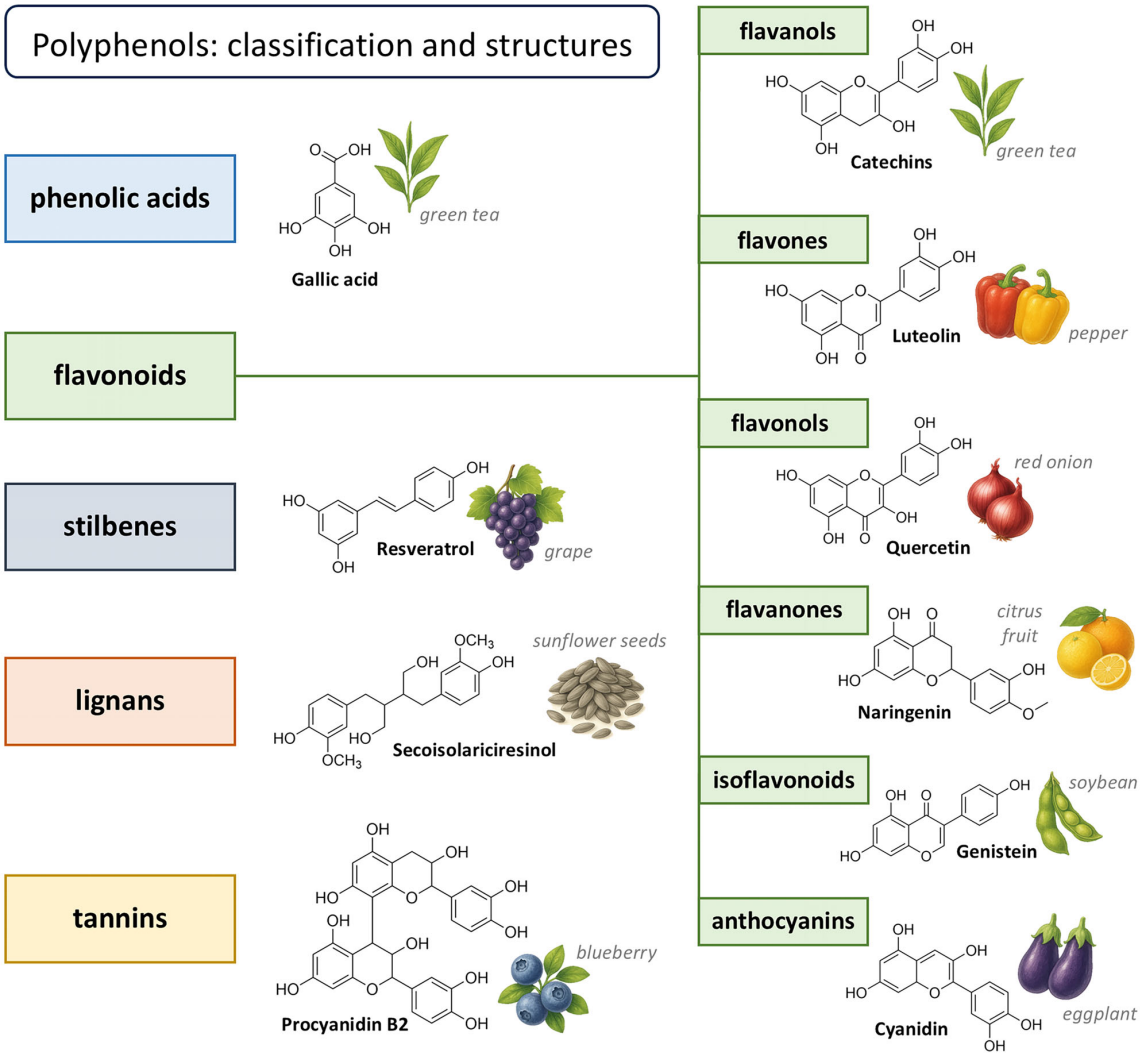
Classification scheme of polyphenols. The structural formulas of some polyphenols belonging to the respective families are shown, along with representations of some organisms in which specific molecules can be found.

Through their metabolism, plants produce a wide range of secondary metabolites, among which polyphenols are particularly noteworthy. These compounds are generally characterized by a structure composed of multiple condensed phenolic rings, one or more hydroxyl groups (─OH), and conjugated double bonds. Polyphenols are ubiquitous in the plant kingdom and are classified into various classes and subclasses based on their chemical structure. The classification is based on 1) the number and arrangement of phenolic groups, 2) the number and position of hydroxyl groups, 3) the types of bonds present between aromatic rings, 4) the degree of polymerization, and 5) the presence of functional modifications. Based on these structural characteristics, polyphenols can be classified into the following groups: phenolic acids, such as caffeic acid (abundant in coffee plants) or gallic acid (found in green tea); flavonoids including quercetin (found in blueberries and other berries); stilbenes such as resveratrol (present in grapes); lignans such as secoisolariciresinol (found in flax, pumpkin, sesame, and sunflower seeds); and tannins including proanthocyanidins. Flavonoids, in particular, are further subdivided into flavanols, flavones, flavonols, flavanones, isoflavonoids, and anthocyanins [13, 14] (Figure 1). Finally, although not technically classified as a polyphenol, curcumin (from *Curcuma longa* L.) is often associated with this group of molecules due to its aromatic structure, plant origin, and chemical and biochemical properties [15].

It is widely recognized that a diet rich in fruits and vegetables is essential for maintaining a healthy lifestyle and that an adequate dietary intake of polyphenols can significantly reduce the risk of developing various degenerative diseases. The protective role of polyphenols is primarily attributed to their ability to modulate and counteract oxidative stress and inflammation, two key factors often involved in the onset and progression of conditions such as Alzheimer's disease, cancer, and cardiovascular disorders [12].

Polyphenols exert their effects through multiple mechanisms, and their effectiveness lies in their pleiotropic nature, that is their capacity to act simultaneously on several molecular pathways. This multifaceted action enhances their therapeutic potential in the prevention and management of complex, multifactorial diseases.

### 2.1 Scavenging Activity of Polyphenols

A direct mechanism by which polyphenols exert their effect is through their role as scavenger molecules, neutralizing free radicals and ROS, such as the hydroxyl radical (•OH), superoxide anion (O_2_^−^•), hydrogen peroxide (H_2_O_2_). Free radicals and ROS are extremely reactive molecules capable of damaging biological macromolecules such as lipids, proteins, and nucleic acids, thereby compromising cellular structures, organs, and tissues. The reactivity of free radicals is primarily due to the presence of unpaired electrons, which drive them to steal electrons from other molecules, disrupting their structure and function. Although O_2_^−^• can be converted into H_2_O_2_ by the enzyme superoxide dismutase (SOD), this compound, in turn, can generate •OH, in the presence of iron through the Fenton reaction.

The phenylic group (the hydroxyl groups attached to aromatic rings) of polyphenols serves as excellent donor of electrons and hydrogen atoms. Upon donating a proton and an electron to a radical, the polyphenol both neutralizes the free radical and forms a phenoxyl radical. This newly formed radical is significantly less reactive, owing to its stabilization through resonance, than the starting radical [13, 16].

The ability to stabilize the unpaired electron via resonance depends on the specific structural features of each polyphenol such as the number and position of conjugated double bonds, the number and arrangement of hydroxyl groups, and the presence of intramolecular hydrogen bonds that further stabilize the phenoxyl radical. Consequently, not all polyphenols exhibit the same scavenging efficiency against ROS and free radicals [17].

### 2.2 Molecular and Cellular Mechanisms of Action of Polyphenols

In addition to neutralizing free radicals and ROS, polyphenols exert their health‐promoting effects by modulating and interacting with various molecular pathways that enable cells to respond to both internal and external stressors, particularly those involving oxidative stress and inflammation.

A great deal of scientific evidence highlights the ability of polyphenols to regulate the nuclear factor erythroid 2‐related factor 2 (Nrf2) signaling pathway, thereby influencing the expression of a wide array of downstream antioxidant response genes [18]. Nrf2 belongs to the Cap'n'Collar family of transcription factors and, under basal conditions, is retained in the cytoplasm bound to the Keap1 protein. This interaction targets Nrf2 for proteasomal degradation via the ubiquitin–proteasome system.

However, in the presence of oxidative stress, cysteine residues on Keap1 become oxidized, inducing conformational changes that disrupt its ability to bind Nrf2. As a result, free Nrf2 translocates into the nucleus, where it binds to Antioxidant response elements (AREs) in the promoter regions of target genes. These include heme oxygenase‐1 (HO‐1), NAD(P)H quinone oxidoreductase, and thioredoxin, among more than 1200 genes activated by Nrf2 [18]. For instance, studies have shown that curcumin treatment of murine JB6 epidermal cells induces HO‐1 expression, an effect that is abolished when, Nrf2 expression is silenced via siRNA. The mechanism through which curcumin activates the Nrf2‐mediated antioxidant response has been investigated, revealing that curcumin binds to Cys151 of Keap1, therefore inhibiting Nrf2 ubiquitination and its subsequent proteasomal degradation [19]. However, it remains unclear whether other polyphenols exert their effects by directly targeting Keap1 in a similar manner. In parallel with the Nrf2 pathway, many polyphenols can modulate the NF‐κB signaling cascade, a key transcription factor involved in inflammation, immunity, cell proliferation, and apoptosis. NF‐kB is typically composed of a p65‐p50 heterodimer [20, 21]. NF‐κB can be activated through multiple signaling pathways often initiated by pro‐inflammatory stimuli such as bacterial lipopolysaccharide (LPS), interleukin‐1β (IL‐1β), and tumor necrosis factor‐α (TNF‐α). These molecules interact with specific membrane receptors, namely TLR, IL‐1R, and TNFR, respectively, initiating intracellular signaling cascades [22].

Under resting conditions, NF‐κB remain sequestered in the cytoplasm through its association with the inhibitor protein κB (IκB). Upon activation by inflammatory stimuli, such as LPS, the IκB kinase (IKK) complex becomes activated and phosphorylates IκB, marking it for degradation via the proteasome. This degradation releases NF‐κB allowing it to translocate into the nucleus where it activates the inflammatory response. For full activation of the p65‐p50 dimer, the p65 subunit needs to be acetylated by the p300/CBP transcriptional coactivators complex [23]. It has been demonstrated that curcumin can directly inhibit IKK activity, thereby preventing IκB phosphorylation and the subsequent nuclear translocation of NF‐κB. The proposed mechanism involves a covalent interaction between curcumin and a cysteine residue (Cys179) in the IKK catalytic domain c. Curcumin, due to its reactive enone structure (α, β‐unsaturated group), can form a covalent bond with Cys179 through a reaction known as Michael addition [24].

Another interesting example is given by resveratrol (RSV) which has also been shown to interfere with the NF‐κB pathway. Resveratrol not only inhibits IKK activity [25] but also disrupts the acetylation of the p65 subunit by p300/CBP [26]. Furthermore, resveratrol enhances the activity of sirtuin 1 (SIRT1), a NAD^+^ dependent deacetylase, which deacetylates p65, thereby reducing its transcriptional activity inflammatory potential [27]. Some authors have highlighted the ability of certain polyphenols to act as inhibitors of cyclooxygenases (COX) [28] or lipoxygenases (LOX), enzymes that play a key role in inflammatory responses, by converting arachidonic acid into prostaglandins and leukotrienes through a multistep process, respectively [28]. Oleacein and oleocanthal, two of the most prominent polyphenols found in extra‐virgin olive oil, known for their strong antioxidant and anti‐inflammatory properties, appear to function as COX inhibitors, showing efficacy comparable to or even greater than that of ibuprofen [29, 30]. In silico modeling analyses suggest that both oleacein and oleocanthal preferentially bind to the active site of COX‐2 over COX‐1, thereby interfering with its enzymatic activity [31]. Beyond the more direct molecular and cellular mechanisms, emerging evidence indicates that polyphenols can also exert beneficial effects indirectly, particularly through interactions with the gut microbiota [32].

Polyphenols have been shown to promote the growth of beneficial bacteria such as those from the *Lactobacillus* and *Bifidobacterium* genus and at the same time inhibiting the proliferation of potentially pathogenic species such as *Clostridium* [32]. Furthermore, the microbiota metabolizes dietary polyphenols, into bioactive compounds that may further contribute to health benefits. These findings come from both in vitro and in vivo studies involving the use of individual polyphenols as well as complex polyphenolic matrices or polyphenol rich foods. This interaction has been associated with various positive outcomes, including slowing the progression of neurodegenerative diseases and reducing inflammation through the production of bioactive molecules [33]. Interestingly, several studies have highlighted a strong connection between polyphenols, the gut microbiota, and skeletal muscle health. Numerous studies conducted on animal models of aging and muscle mass loss have shown that the intake of certain polyphenols can have a protective effect on muscle tissue contributing to enhanced muscle strength and performance. Although to a lesser extent, these findings have also been supported by some studies conducted on humans. However, the beneficial effects of polyphenol intake exhibit significant interindividual variability, largely influenced by differences in the taxonomic composition and metabolic activity of the intestinal microbial communities [34].

## 3 Muscle Cell Proliferation and Differentiation: A Role for Polyphenols

Myogenesis is the biological process through which skeletal muscle fibers are formed from undifferentiated progenitor cells. This highly regulated sequence includes several key phases: activation, proliferation, differentiation, and fusion of satellite cells. These muscle‐resident stem cells are activated in response to stimuli such as physical exercise, muscle damage, or specific hormonal signals.

Skeletal muscle development begins during the embryonic and foetal stages and is completed during the first weeks and months of postnatal life. At birth, myofibers are not yet fully specialized: during early postnatal development, in response to nerve stimuli, movement, and mechanical loading, they differentiate into slow‐twitch and fast‐twitch fibers. Muscle stem cells (MuSCs), already present at birth but undergoing maturation, begin to occupy the space between the basal lamina and the sarcolemma.

**FIGURE 2:**
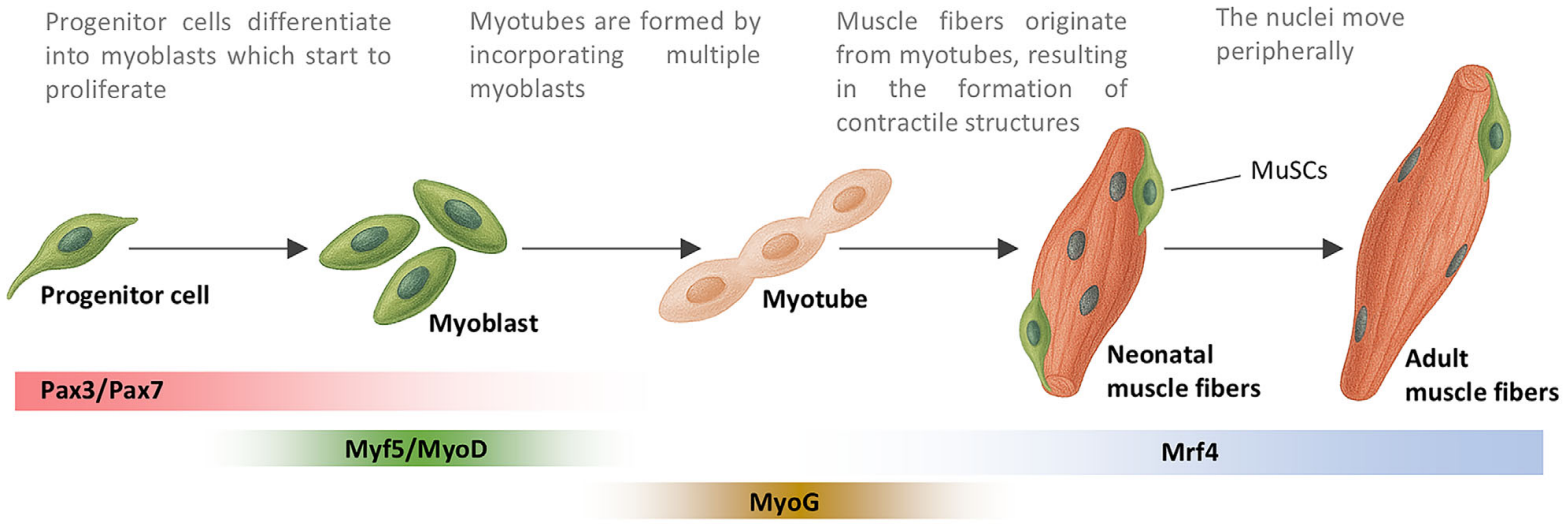
Simplified representation of the development of a multinucleated muscle cell with some important factors involved.

At this stage MuSCs represent a higher proportion of muscle cells compared to adulthood, providing significant potential for muscle growth, development, and remodeling. Unlike mature muscle fibers, neonatal fibers are characterized by centrally located nuclei which migrate peripherally as development progresses. Finally, neuromuscular junctions, although present at birth, continue to mature postnatally with, improvements in motor neurons myelination and consequently enhancing contractile activity. In adulthood, skeletal muscle fibers are relatively stable, performing their tasks related to movement, posture, protection of bones and internal organs, and thermoregulation. However, skeletal muscle homeostasis remains responsive to both internal and external stimuli. Positive environmental factors, such as physical exercise can promote muscle remodeling and improve functional capacity. Conversely, negative influences including sedentary behavior, inadequate nutrition or other unhealthy lifestyle factors, can contribute to muscle atrophy and the development of pathological conditions [35]. The development and remodeling of skeletal muscle are governed by a complex network of molecular signals and a tightly sequence of transcription factors activation and developmental pathways. During embryogenesis, myogenesis is orchestrated by key myogenic regulatory factors (MRFs) including Pax3, Pax7, Myf5, MyoD, Mrf4, and MyoG. These transcription factors coordinate the proliferation, differentiation, and fusion of myoblasts into mature muscle fibers. Specifically, Pax3 and Pax7 are fundamental in the initial stages for the identity of dermomyotomal progenitors. Myf5 and MyoD are activators of the myogenic lineage, and Mrf4 and MyoG are important during the differentiation phase and the formation of multinucleated fibers (Figure 2).

**FIGURE 3:**
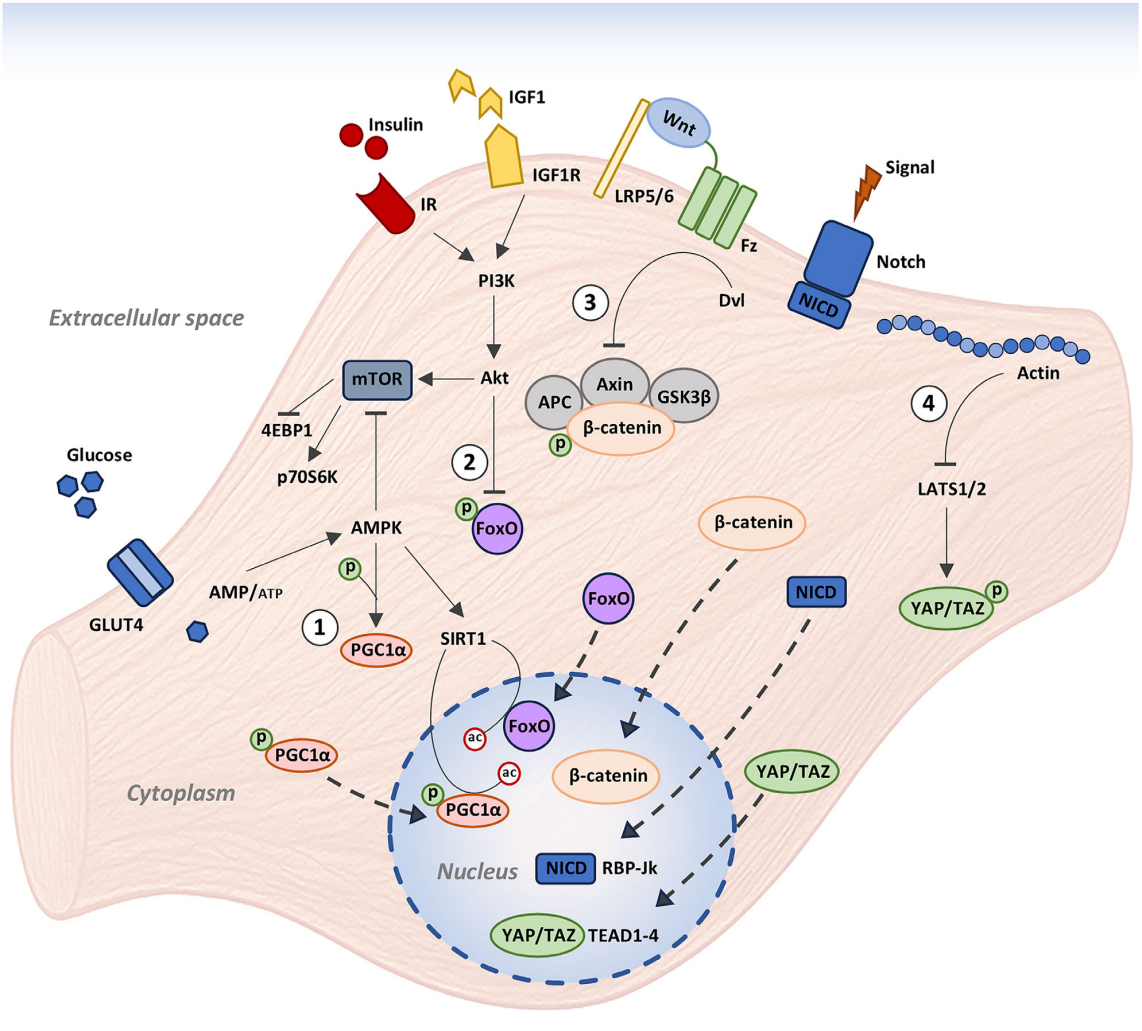
Simplified representation of the main molecular and cellular pathways involved in the development, regeneration, and maintenance of a muscle cell. (1) AMPK phosphorylates PGC1α, which, once in the nucleus, can be deacetylated by SIRT1. (2) Akt inhibits the dephosphorylation of FoxO. Dephosphorylated FoxO enters the nucleus where it can be deacetylated by SIRT1. (3) In the absence of a Wnt signal, β‐catenin is degraded via the proteasome by the APC/Axin/GSK3β complex. When Wnt binds to the Frizzled and LRP5/6 receptors, the protein Dvl (Disheveled) is activated. Dvl inhibits the APC/Axin/GSK3β complex, preventing the degradation of β‐catenin. This allows β‐catenin to accumulate in the cytoplasm and then translocate into the nucleus, where it activates transcription of target genes. (4) Actin filaments indicate strong mechanical tension and cell adhesion. Actin accumulation can inhibit the activity of LATS1/2, promoting the activation of YAP/TAZ. LATS1/2 (Large Tumor Suppressor Kinases) are key kinases in the Hippo pathway. When activated, they phosphorylate YAP/TAZ, causing their retention in the cytoplasm and degradation via the proteasome. If not phosphorylated by LATS1/2, YAP/TAZ translocate into the nucleus, bind to cofactors such as TEAD, and activate genes involved in proliferation, survival, and regeneration.

During the continuous remodeling of the muscle fibers, characteristic of the adult skeletal muscle, one of the central molecular pathways in the process is PI3K/Akt/mTOR, which promotes muscle growth through the activation of protein synthesis. In particular, Akt activated by growth factors such as IGF‐1 (Insulin‐like Growth Factor 1), phosphorylates and inhibits TSC2, thereby releasing mTORC1, which in turn promotes protein translation by activating S6K1 and inhibiting 4E‐BP1. This pathway is also critical for the survival, proliferation, and differentiation of satellite cells [35]. In addition, the Wnt/β‐catenin signaling pathway contributes to the activation and differentiation of satellite cells, whereas the Notch signaling pathway plays an opposite role by maintaining satellite cells in a quiescence state [36, 37]. During muscle atrophy, the transcription factor FoxO3, similar to FoxO1, is activated under conditions of physical inactivity, malnutrition, or disease. Once activated, FoxO3 regulates the expression of several genes, including FBXO32 (Atrogin‐1) and TRIM63 (MuRF1), which in turn control the ubiquitin–proteasome pathway responsible for protein degradation [38]. Another critical regulator of muscle cell function is AMP‐activated protein kinase (AMPK), a key energy sensor that plays a central role in maintaining energy homeostasis. AMPK is activated when the cellular AMP/ATP ratio increases, as occurs during energy stress such as physical exercise or hypoxia. Upon activation, AMPK promotes ATP synthesis, enhances glucose uptake via GLUT4 transporters, increases fatty acid beta‐oxidation, and stimulates mitochondrial biogenesis [39]. Finally, mechanical loading of muscle tissue generates mechanical stress perceived through the extracellular matrix, the plasma membrane, and adhesion complexes. This mechanical signal activates integrins and subsequently focal adhesion kinase (FAK), initiating a signaling cascade that modulates the activity of the YAP/TAZ complex, transcriptional co‐activators capable of translocating to the nucleus, where they activate downstream gene transcription and promote muscle growth and regeneration [40] (Figure 3).

Polyphenols, have demonstrated potential in modulating myogenesis, thereby influencing muscle growth and repair. Evidence from the current literature, suggests that polyphenol supplementation may serve as a promising strategy to support muscle cell health through multiple mechanisms. In a study conducted on Wistar rats supplemented with oligomeric proanthocyanidins (PCOs) derived from grape seeds for 2 weeks before and after the induction of muscle contusion, an acceleration in satellite cell activation, and enhanced muscle regeneration were observed. Additionally, PCO treatment reduced neutrophil infiltration and promoted an earlier transition of macrophages toward an anti‐inflammatory M2 phenotype accompanied by increased levels of the cytokine IL‐10 [41]. In addition, numerous other studies further suggest that polyphenols can modulate mitochondrial function [42], increase muscle performance by increasing cyclic AMP (cAMP) levels [43], and mitigate age‐related muscle mass loss (sarcopenia) [44, 45].

### 3.1 The Role of Oxidative Stress and Inflammation in Muscle Development and Differentiation

**FIGURE 4:**
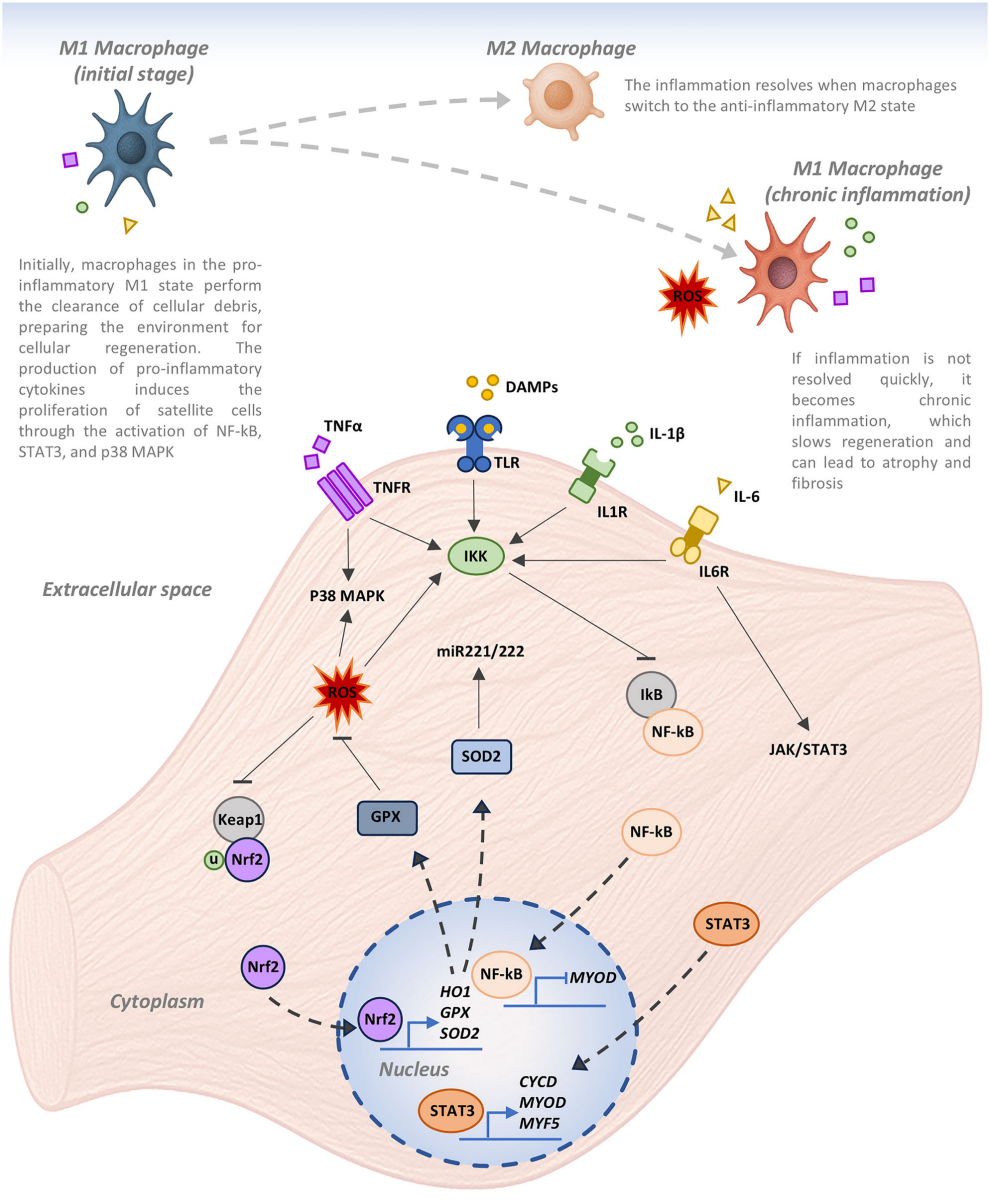
Simplified representation of the action exerted by ROS and inflammation on certain cellular pathways.

As might be expected, oxidative stress and inflammation are two critical factors that significantly influence the development, regeneration, and remodeling of muscle cells, leading to either physiological adaptations or pathological conditions (Figure 4).

Under physiological conditions, low concentrations of ROS actively promote muscle cell differentiation and the activation of satellite cells. At these low levels, ROS stimulate transcription factors such as Nrf2 and NF‐κB, which in turn induce the expression of antioxidant enzymes including heme oxygenase‐1 (HO‐1), glutathione peroxidase (GPX), and superoxide dismutase 2 (SOD2). SOD2 activity promotes the proliferation of satellite cells through the induction of miR‐221/222, which inhibit the expression of the p57 gene thereby promoting satellite cell activation and expansion into myoblasts. In a subsequent phase, GPX‐1 supports myogenic differentiation by maintaining low oxidative stress levels and reducing the inhibitory effect of NF‐κB on MyoD, thus enabling myotube formation.

In contrast, excessive ROS levels exert deleterious effects by damaging DNA, proteins, and membranes, negatively impacting satellite cells, myoblasts, and mature myofibers. In detail, high ROS levels impair satellite cell function by inhibiting their proliferation and regenerative. In pathological context, ROS overproduction can induce senescence and apoptosis in muscle stem cells. In differentiating myoblasts, chronic ROS exposure leads to persistent activation of NF‐κB, which suppresses MyoD expression, and inhibits differentiation. Moreover, ROS interfere with the expression of miR‐206, a microRNA crucial for myotube formation. Finally, in mature myofibers, ROS excess results in mitochondrial dysfunction, increased apoptosis, and ultimately muscle atrophy [46].

Similarly, in the context of inflammation, a controlled low‐grade inflammatory response, often referred to as “beneficial” inflammation, plays a key role in muscle regeneration. In this phase, macrophages are initially polarized to the M1 phenotype, promoting the release pro‐inflammatory cytokines such as interleukin‐ 1β (IL‐1β) and interleukin‐6 (IL‐6), which activate quiescent satellite cells, inducing them to proliferate. Indeed, studies using primary myoblasts and C2C12 cells, have demonstrated that 5 days after barium chloride‐induced muscle injury, the expression levels of IL‐1β and IL‐6 increased by an approximately 20‐fold. Furthermore, treatment of uninjured C2C12 cells with IL‐1β on lead to a dose‐dependent increase in the proliferative rate, an increase in the transcriptional levels and protein expression of IL‐6, and enhanced NF‐κB activity. Comparable effects were observed following TNF‐α treatment [47]. Following the resolution of the M1 phase, macrophages polarization shifts to the M2 phenotype, characterized by the release of anti‐inflammatory factors that support myogenic differentiation and the resolution of inflammation. To investigate this transition, an in vivo model of muscle atrophy was used to assess the regenerative effects of macrophage activated with macrophage colony‐stimulating factor (M‐CSF) on the atrophied soleus muscles. The findings revealed both an increase in muscle fiber diameter and accelerated recovery of muscle strength in M‐CSF‐treated samples compared to controls. Furthermore, co‐culture experiments using atrophied myoblasts and M‐CSF‐activated macrophages confirmed the acquisition of an anti‐inflammatory M2‐type expression profile and phenotype in these macrophages [48].

However, if the inflammatory state persists over time, resulting in chronic “pathological” inflammation, macrophages remain predominantly in the M1 activation state. This persistence exacerbates the inflammatory response, impairs satellite cell function by maintaining them in a non‐functional state and ultimately contribute to the development of fibrosis [49]. Notably, activation of the NF‐κB pathway in skeletal muscle has been shown to be sufficient to induce muscle atrophy, while its inhibition appears to attenuate inflammatory process in muscle cells [50]. On the other hand, sustained activation of NF‐κB is correlated with various skeletal muscle pathologies, and muscle mass loss is a common feature in chronic inflammatory conditions [51]. Typical markers of inflammation such as TNF‐α and IL‐1β are also markers of cachexia [52], and muscle biopsies from patients with severe cachexia are characterized by high NF‐κB activation [53].

### 3.2 Polyphenols as Modulators of Oxidative Stress and Inflammation in Muscle Cells

Given the critical role of oxidative stress and inflammation in the development and differentiation of muscle cells and considering the well‐established antioxidant and anti‐inflammatory properties of polyphenols, it is reasonable to hypothesize that these bioactive molecules may influence the redox states of muscle cells.

Numerous studies support the beneficial effects of polyphenols on muscle physiology, particularly their ability to mitigate the detrimental consequences of excessive oxidative and inflammatory stress. In this review, we summarized key findings from both in vitro and in vivo studies investigating the effects of individual polyphenols, their combinations or mixtures, and crude or enriched extracts derived from polyphenol‐rich sources.

One commonly method to induce oxidative stress in vitro involves the administration of H_2_O_2_ at varying concentrations. While low concentrations of H_2_O_2_, are known to function as a positive stimulus, essential for cellular development, it is well‐documented that exposure beyond a certain threshold, induces oxidative stress, thereby posing risks to cellular macromolecules and structures.

For instance, Bosutti and colleagues, examined the effects of RSV on murine C2C12 muscle cells, administered at different concentrations (10, 20, 40, 60 µM) for 24‐48 h, and exposed to escalating concentrations of H_2_O_2_ (100, 500, 1000 µM) to simulate oxidative stress. As expected, low concentrations of ROS increased myoblast migration, while high concentrations were detrimental. Interestingly, RSV exhibited a similar dose‐dependent response. High doses of RSV, in fact, negatively affected several cellular parameters, whereas lower doses (particularly 10 µM) had beneficial effects. In particular, while RSV did not appear to enhance the metabolic state of myoblasts or the oxidative capacity of myotubes, it was effective in counteracting H_2_O_2_‐induced impairments in cell migration and in preserving the activity of myosin ATPase, an enzyme that hydrolyses ATP and plays a crucial role in muscle contraction and cell motility. Conversely, it had no protective effects regarding cell fusion, showed negative effects regarding mitochondrial damage, and even exacerbates the adverse effects of H_2_O_2_ regarding the metabolic state of myoblasts or the oxidative capacity of myotubes [54]. A similar dose‐dependent pattern was reported by Zhang and colleagues [55], who treated bovine skeletal muscle cells (BMCs) with H_2_O_2_ to induce oxidative stress alongside increasing concentrations of RSV (0–90 µM). They observed that RSV at 30 µM enhanced cell viability and decreased ROS production likely through modulation of the Nrf2 and SIRT1 signaling pathways.

The Nrf2 signaling pathway and its downstream target genes, such as HO‐1, are modulated by phloroglucinol, a compound extracted from the brown alga *Ecklonia cava* known for its diverse pharmacological activities. Treatment with phloroglucinol confers protection against oxidative damage in C2C12 cells exposed to H_2_O_2_ by reducing ROS levels and decreasing cell death. Additionally, it mitigates DNA damage as evidenced by reduced phosphorylation of the histone marker γH2AX. Interestingly, phloroglucinol also significantly decreases cell viability at concentrations ≥40 µg/mL [56].

In contrast, piceatannol, a RSV derivative found in grapes or blueberries, does not appear to exert toxic effects even at higher concentrations. When administered to C2C12 cells, at concentration ranging from 10–50 µM, under H_2_O_2_‐induced oxidative stress, picetannol strongly reduced ROS accumulation and dose‐dependently induced the expression of HO‐1 and SOD1, suggesting superior protective effects against oxidative stress compared to other analogous, including RSV [57]. Collectively, these findings suggest that although polyphenols, often exhibit similar antioxidant outcomes, each compound possess specific characteristic in terms of efficacy, tolerability, and mechanisms of action. For example, Cuijpers and colleagues observed that hesperetin, a flavanone mainly found in citrus fruits such as lemons, limes, and mandarins, but not ellagic acid, modulates the expression of myosin heavy chain and promotes the fusion of C2C12 cells under oxidative stress [58]. An increasing number of studies have investigated the effects of polyphenols administered as mixture, in combination with other molecules or directly as crude juices and extracts. This approach more closely mimics real‐life dietary conditions, where multiple bioactive compounds interact synergistically or antagonistically. Although it complicates the identification of individual compounds responsible for specific effects, it offers a practical framework for animal or human studies. Additionally, it supports the development of effective interventions at lower costs, by avoiding the need to isolate and purify single molecules. Synergistic interactions may also allow the use of each molecule at lower concentrations, potentially well below toxic thresholds, thereby improving both safety and efficacy. In this context, grape stems extracts have demonstrated substantial protective effects against oxidative stress in vitro in muscle cells. The study by Capozzi and colleagues [59] examined the effects of polyphenolic extracts from grape skins and seeds (c.v. Syrah), harvested at different ripening stages, using human skeletal muscle cells stimulated with palmitic acid to induce oxidative stress. All extracts reduced lipid peroxidation by 44%–60%, increased HO‐1 expression by 75%–132%, and improved mitochondrial activity by 47%–68%. Interestingly, only extracts from seeds harvested at earlier ripening stages improved insulin sensitivity by 50%, while the others showed no significant effects. Polyphenolic profiling via Folin–Ciocalteu and DPPH assays revealed higher antioxidant activity in immature berries underscoring how extract composition and efficacy are influenced by both plant part and maturation stage. Additionally, the grape cultivar used plays a significant role in determining the total and individual polyphenol contents. Extracts from three different cultivars (Mavrotragano, Mandilaria, and Moshomavro) showed distinct polyphenolic profiles [60]. Among them, the Moshomavro extract, with the weakest DPPH and ABTS scavenging activity showed no significant effect on ROS levels, protein carbonylation, GSH content, or lipid peroxidation in C2C12 muscle cells. Conversely, extracts from Mavrotragano and Mandilaria cultivars, showed comparable scavenging capacities, and significantly improved all evaluated parameters except GSH, where only the Mavrotragano extract produced a statistically significant change compared to the control. These findings suggest that both the total polyphenol content and the profile and quantities of each single bioactive molecule are important. Contrasting results were obtained with extracts of different cultivars of *Moringa oleifera*, a Brassicales plant widely known for its therapeutic properties. Tested on C2C12 muscle cells exposed to H_2_O_2_, extracts from various cultivars exhibited similar antioxidant effects despite metabolic differences in glucosinolates, flavonoids, phenolic acids, and other minor metabolites as revealed by UHPLC/QTOF‐MS analysis [61]. These extracts protected against oxidative stress through modulation of SIRT1‐PPARα and Nrf2 pathways, and regulation of HO‐1, CAT, SOD, GPx, and GST enzymatic activities [62, 63], with no statistically significant differences among cultivars.

Given that oxidative stress and inflammation often coexist, numerous studies have investigated the anti‐inflammatory potential of polyphenols in muscle pathophysiology. Polyphenols have been tested both as isolated bioactive molecules or in combination with each other or with other molecules, yielding promising data regarding their role in preventing or alleviating inflammation‐related muscle damage. Compounds such as RSV, rutin, and complex extracts made from *Lonicera caerulea* and *Morus alba* (mulberry) berries have been studied for their dual antioxidant and anti‐inflammatory properties. RSV, for instance, effectively suppresses palmitate‐induced inflammation in skeletal muscle cells by inhibiting NF‐κB, independently of SIRT1 activation [64]. Another study reported that RSV prevents TNF‐α‐induced muscle atrophy by modulating the Akt/mTOR/FoxO1 pathway, thus promoting the maintenance of muscle mass [65].

Rutin, a bioflavonoid commonly found in plants of the Rutaceae family, also exhibit anti‐inflammatory activity. In LPS‐stimulated C2C12 cells, rutin reduced the expression of pro‐inflammatory cytokines such as TNF‐α and IL‐6 likely through NF‐κB inhibition supporting its protective role in muscle inflammation [66]. Polyphenols‐rich extracts have also shown efficacy in animal models, suggesting potential for translational applications. For example, *Lonicera caerulea* berry extracts, alleviated muscle fatigue and inflammation in murine models subjected to intense physical exercise. These extracts reduced oxidative stress, apoptosis, and promoted muscle regeneration. Mechanistically they influenced multiple signaling pathways, including AMPK‐PGC‐1α‐NRF1‐TFAM (involved in mitochondrial biogenesis), miRNA‐133a/MyoD/MyoG/IGF‐1R, (regulating muscle proliferation, differentiation), and PI3K/Akt/mTOR. Similarly, *Morus alba* fruit extract was evaluated in a murine model of obesity‐induced muscle. Bioinformatic molecular docking analysis further supported the functional relevance of these pathways, revealing a strong interaction between cyanidin‐3‐glucoside, a principal bioactive component in the extract, and the AMPK binding site [67]. Finally, *Morus alba* fruit extract was evaluated in a murine model of obesity‐induced muscle inflammation and mitochondrial dysfunction. The extract attenuated inflammatory response by regulating miR‐21, miR‐132, and miR‐143, microRNAs involved in inflammatory processes, and activating the AMPK/SIRT axis, ultimately improving mitochondrial function [68]. Overall, these findings support the notion that polyphenols exert multifactorial actions in muscle tissues, with potential to modulate key oxidative and inflammatory signaling pathways, thus offering promising avenues for therapeutic intervention.

### 3.3 Polyphenols and Their Impact on Mitochondrial Activity in Muscle Cells

Oxidative stress and mitochondrial dysfunction are often related concepts. Mitochondria, after all, represent the “powerhouses” of cells, responsible for producing ATP through oxidative phosphorylation. During this process, a small percentage of molecular oxygen (O_2_) undergoes incomplete reduction resulting in the generation of ROS. During intense muscular exertion, energy demand increases, leading to increased mitochondrial activity, and consequently elevated ROS production. Skeletal muscle cells require high amounts of ATP for contraction, especially during intense or prolonged physical activities. Metabolic adaptation to aerobic training enhances both the number and size of mitochondria within muscle cells a process known as mitochondrial biogenesis, which contributes to improved endurance, reduced lactic acid accumulation, and faster recovery. Moreover, mitochondria regulate lipid and glucose metabolism and participates in crucial cellular processes including signaling, cell cycle regulation and apoptosis.

In this context, RSV has been widely studied for its effects on mitochondrial function particularly in skeletal muscle tissue. Several studies have explored its role as a modulator in mitochondrial biogenesis and function, noting effects analogous to those induced by physical exercise and counteracting dysfunctions related to age, obesity, and inactivity. One of the investigated but still debated mechanisms involves the regulation of SIRT1 in RSV‐mediated mitochondrial biogenesis and function under stress conditions. Menzies and colleagues [69] demonstrated that, in skeletal muscle cells, RSV increases SIRT1 expression, while chronic contractile activity (CCA) fails to do so. Conversely, CCA upregulates PGC‐1α expression, potentially through p38 and AMPK phosphorylation pathways, effects not replicated by RSV alone. Interestingly, combined treatment with RSV and CCA elevated the expression of both SIRT1 and PGC‐1α, suggesting a potential synergistic effect. In Sirt1‐knockout (KO) mice reduced mitochondrial content, and functionality, increased fatigue, and disrupted glucose homeostasis was observed. However, these phenotypes were only evident during high energy demand and sustained contractile activity. Both ROS levels and mitochondrial respiratory parameters were improved by RSV, exercise, or their combination in Sirt1‐KO mice, suggesting that at least some of the RSV effects occur independently of SIRT1.

The essential role of SIRT1 in mediating RSV mitochondrial effects was further supported by Price and colleagues [70], who showed that moderate doses of RSV increased mitochondrial biogenesis, AMPK activation, and NAD^+^ levels in wild‐type mice but not in Sirt1‐KO mice. High doses of RSV did activate AMPK but independently of SIRT1. In any case, regardless of the dose used, no improvements in mitochondrial function were observed in Sirt1‐KO mice, indicating that SIRT1 is crucial for certain mitochondrial benefits conferred by RSV.

Beyond SIRT1, Huang and colleagues [71] explored the role of RSV in preventing muscle mass loss, using both an in vivo model of obesity‐induced sarcopenia and an in vitro cellular system RSV administration reduced muscle atrophy and enhanced mitochondrial biogenesis, function and respiratory capacity while also mitigating oxidative stress. These protective effects were abolished by the silencing of PKA, LKB1, and AMPK, via siRNA transfection, emphasizing the importance of these signaling pathways in the mechanism of action of RSV. Similar findings have been reported by other studies using comparable experimental models, all pointing to confirm the potential of RSV for enhancing mitochondrial integrity and regenerative capacity in skeletal muscle [72, 73].

In addition to RSV, other polyphenols have demonstrated similar protective effects. Tyrosol, abundant in extra‐virgin olive oil, has shown activity against dexamethasone‐induced muscle atrophy [74] while gallic acid, has been found to attenuate exercise‐induced muscle damage by inhibiting mitochondrial oxidative stress and ferroptosis, a form of regulated cell death associated with lipid peroxidation [75]. Beyond individual polyphenols, complex polyphenols mixtures or crude polyphenols rich extracts have also exhibited beneficial effects on mitochondrial function. For instance, sugarcane top ethanol extract (STEE), was shown to upregulate the transcriptional levels of PGC‐1α in C2C12 cells. Transcriptomic analysis revealed that STEE treatment altered the expression of genes involved in mitochondrial function, fatty acid metabolism, mitogen‐activated protein kinase (MAPK) and cAMP signaling, as well as inflammatory cytokine production.

Additionally, STEE treatment increased mitochondrial membrane potential, further supporting its bioactivity [76]. As previously discussed, H_2_O_2_, induces oxidative stress leading to apoptosis and mitochondrial damage in C2C12 cells. Cells treated with 100 µM H_2_O_2_ displayed elevated apoptosis and severely compromised mitochondrial structure. However, the administration of a flavonoid‐rich cocoa extract (CPE) at 10 µg/mL, refreshed every 24 h, offered substantial defence protecting cells from mitochondrial damage and death in both early and late stages of differentiation. Treated cells exhibited a greater number of mitochondria with improved morphology and more developed cristae, both during early and late stages of differentiation [77].

When working with complex crude extracts, the method of extraction is crucial, as it significantly influences the bioactive composition and, consequently, the biological activity. The solvent used (e.g., aqueous vs. organic) can result in extracts with differing polyphenol profiles and antioxidant properties, thereby differentially affecting mitochondrial functionality [78]. This highlights the importance of standardizing extraction protocols and thoroughly characterizing the chemical composition when evaluating polyphenol‐rich extracts for biomedical applications.

### 3.4 The Effects of Polyphenols on the Metabolism of Muscle Cells

Insulin resistance is a metabolic phenomenon in which cells, including those in muscle, liver and adipose tissue, exhibit a diminished response to insulin. Under normal physiological conditions, elevated blood glucose levels trigger insulin secretion, which promotes glucose uptake into cells.

This uptake is mediated by the binding of insulin to its receptor leading to the phosphorylation and activation of insulin receptor substrates (IRS). Activated IRS, in turn stimulates the PI3K/Akt signaling cascade which plays a central role in regulating cellular glucose uptake via GLUT4 translocation, glycogen synthesis through GSK3β inhibition and glycogen synthase (GS) activation, and protein synthesis via mTOR signaling.

However, in the presence of oxidative stress, inflammation, or the accumulation of intracellular lipids IRS becomes phosphorylated on serine residues rather than tyrosine residues, thereby impairing its function. This disruption is a hallmark of insulin resistance, a condition that can ultimately lead to type 2 diabetes, non‐alcoholic fatty liver disease, metabolic syndrome, and cardiovascular complications.

In this context, AMPK and sirtuins, such as SIRT1, also merge as crucial modulators of insulin signaling and cellular metabolism. AMPK enhances GLUT4‐mediated glucose uptake, inhibits fatty acid synthesis, and promotes lipid oxidation. SIRT1, meanwhile, deacetylates and activates PGC‐1α and positively modulates IRS function [79].

It is well known that physical exercise significantly influences muscle cell metabolism [80]. A reduction in ATP levels during exercise, increase the AMP/ATP ratio, thereby activating AMPK. Additionally, prolonged physical activity leads to the activation of SIRT1, Akt, PGC‐1α, and the mTOR pathway collectively contributing to improved metabolic homeostasis. Beyond the metabolic benefits of exercise, the administration of polyphenols, as single purified molecules or in the form of complex mixtures, has been increasingly recognized as a promising strategy to improve skeletal muscle metabolism and counteract insulin resistance. Among the most extensively studied polyphenols in this context is certainly RSV [81, 82, 83, 84, 85], alongside other notable compounds such as quercetin [81], rosmarinic acid [86, 87], and complex extracts from *Phyllanthus niruri* [88], black tea [89], and lotus leaves [90].

Both quercetin and RSV have demonstrated the capacity to activate the AMPK pathway. In human myoblasts, these polyphenols increased AMPK phosphorylation improved insulin signaling, promoted glycogen synthesis via GSK3β modulation, and reduced lactate accumulation [81].

Furthermore, RSV enhanced myogenin expression and modulated the activity of SIRT1, AMPK, PP2A, and key components of the mTOR pathway in C2C12 cells cultured under high‐glucose conditions. These changes were accompanied by a shift toward faster circadian rhythms, as evidenced by decreased levels of pBMAL1 and CRY1 [82]. Insulin resistance is also closely linked to obesity and elevated plasma levels of free fatty acids (FFAs). High FFA levels, such as those induced by palmitate, can impair insulin signaling in skeletal muscle. Studies using GLUT4myc overexpressing rat myoblasts have shown that RSV reverses palmitate‐induced disruptions in IRS‐1, the mTOR pathway, AMPK, and GLUT4, effectively restoring glucose uptake and insulin sensitivity [83, 84]. Similar results were observed in mice fed a high‐fat diet (HFD) and treated with RSV, which led to reduced body weight gain, improved glycaemic control, and decreased lipid accumulation [85].

Other polyphenols exhibit comparable effects. Rosmarinic acid, for example, reversed the negative effects of palmitate on IRS‐1, Akt, mTOR, and GLUT4, restoring insulin‐stimulated glucose uptake [86]. It also promoted mitochondrial biogenesis through the activation of AMPK, PGC‐1α, and SIRT1 [87]. Extract from *Phyllanthus niruri*, a tropical plant commonly found in coastal areas, increased glucose uptake and reduced ROS levels in palmitate treated C2C12 cells suggesting a synergistic antioxidant and metabolic modulation [88]. The interaction between polyphenols and physiological stimuli such as physical exercise, further highlights their therapeutic potential. For instance, black tea extract, when administered alongside physical training, significantly improved exercise endurance, AMPK activation, and GLUT4 translocation, amplifying the beneficial effects of exercise alone [89].

A compelling example of the protective role of polyphenols is provided by a polyphenol‐rich water extract from lotus leaves (*Nelumbo nucifera* Gaertn), tested in a murine model of dexamethasone‐induced sarcopenia. The extract improved motor performance and grip strength downregulated the expression of atrogin‐1, MuRF1, and FoxO3a, and promoted anabolic mTOR signaling, demonstrating not only metabolic improvements but also muscle‐preserving properties [90].

Overall, these studies strongly support the role of polyphenols as modulators of muscle metabolism and insulin sensitivity, through activation of AMPK and positive regulation of insulin‐dependent signaling pathways. Therefore, polyphenols can be considered promising nutraceuticals for the prevention and management of insulin resistance and related metabolic disorders.

Oxidative stress, inflammation, mitochondrial activity, and metabolism are therefore influenced by specific mechanisms and factors, either unique or shared among them, all integrated to elicit coordinated responses. In this context, epigenetics and its regulation play a central role in modulating and integrating these responses. Although the impact of epigenetics on numerous biological processes has long been recognized, only recently has evidence been accumulating regarding the influence of certain polyphenols on epigenetic processes and their regulation. In a recent comprehensive review, Rodrigues and colleagues [91] address this topic, highlighting the role of epigenetics as a “bridge” for modulating various aspects of aging and muscle function. According to the authors, polyphenols can modulate epigenetic responses through interactions with key enzymes such as DNA methyltransferases (DNMTs), histone deacetylases (HDACs), and sirtuins (particularly SIRT1), regulating downstream pathways with effects on numerous biological processes including oxidative stress, inflammation, mitochondrial function, energy metabolism, proteostasis, and autophagy.

## 4 Polyphenols and Skeletal Muscle Diseases

Up to this point, we have analyzed the multifaceted impact of polyphenols on key physiological processes in skeletal muscle cells, including oxidative stress, inflammation, mitochondrial function, and metabolic regulation and as evidenced by the data discussed, these processes are clearly intricately interconnected. Disruption in one often propagates to others, collectively contributing to a spectrum of pathological conditions affecting skeletal muscle. These include myopathies, insulin resistance, type 2 diabetes, metabolic syndrome, sarcopenic obesity, muscle steatosis, sarcopenia, and cachexia [92]. To summarize, mitochondrial dysfunction in skeletal muscle is a hallmark of numerous chronic, metabolic, inflammatory, and degenerative disease. Under physiological conditions, mitochondria maintain energy homeostasis through substrate oxidation and controlled ROS production, which also function as signaling molecules in various intracellular pathways. However, chronic redox imbalance, leads to excessive mitochondrial ROS generation, which, damages mitochondrial DNA, impairs membrane integrity, and inhibits respiratory chain enzyme activity [46]. This results not only in diminished oxidative capacity but also in the establishment of a pro‐inflammatory microenvironment, characterized by sustained activation of transcription factors such as NF‐κB and increased secretion of TNF‐α and IL‐6 [49, 50, 51, 52, 53]. These cytokines, in turn, impair insulin signaling pathways [93], contributing to a vicious cycle of metabolic dysfunction.

In parallel, ongoing mitochondrial impairments disrupt β‐oxidation of fatty acids, favoring the accumulation of lipotoxic intermediate such as diacylglycerols and ceramides. These compounds exacerbate inflammation and insulin resistance [94]. Such mechanisms are particularly relevant in the pathogenesis of metabolic sarcopenia and sarcopenic obesity, where muscle mass reduction is coupled with adverse metabolic profile [95]. Therefore, oxidative stress, chronic low‐grade inflammation, mitochondrial dysfunctions, and metabolic imbalances collectively represent synergistic triggers that drive the progressive deterioration of skeletal muscle function.

Another important, though often overlooked aspect impacting skeletal muscle health is the degeneration of motor neurons and neuromuscular junctions. Although this phenomenon originates in the nervous system, it directly affects skeletal muscle by impairing neuromuscular transmission and leading to reduced muscle activation. Over time, this contributes to muscle atrophy altered gene expression and metabolic dysregulation in muscle tissue [96]. Aging and neurodegenerative diseases, such as sarcopenia and amyotrophic lateral sclerosis (ALS), are marked by progressive neuromuscular dysfunction. These conditions are characterized by muscle atrophy and structural degeneration of neuromuscular junctions. In this context, polyphenols have emerged as potential neuroprotective agents. Preclinical studies, particularly in murine models suggest that specific polyphenolic classes of including flavonoids, anthocyanins, and stilbenes, may help preserve neuromuscular health.

A study conducted on C57BL/6J mice demonstrated that diets enriched with flavonoids derived from green tea or cocoa mitigated age‐related neuromuscular decline. Both treatments showed beneficial effects on animal survival, reduced the presence of histological markers of muscle degeneration such as central nuclei and lipofuscin aggregates, and increased satellite cell density [97]. At the level of neuromuscular junctions, both treatments promoted reinnervation and enhanced synaptic maturity. Interestingly, tea‐derived flavonoids increased VAChT and VGluT2 afferent synapses and reduced pro‐inflammatory microgliosis, while cocoa‐derived polyphenols increased VGluT1 afferent synapses, suggesting mechanistic differences likely attributable to their distinct polyphenol compositions [97]. In ALS, a neurodegenerative condition characterized by the loss of motor neurons and resultant skeletal muscle atrophy, oxidative stress and inflammation are central to the disease progression. In this context, protocatechuic acid (PCA), a metabolite of anthocyanins contained in blackberries and bilberries, showed neuroprotective effects in a G93A‐SOD1 transgenic mouse model of ALS expressing a mutated form of human superoxide dismutase 1. PCA treatment delayed disease progression preserved muscle strength and neuromuscular integrity and reduced neuronal apoptosis and neuroinflammation in the spinal cord [98].

Conversely, RSV, despite its well‐documented antioxidant, anti‐inflammatory, and metabolic effects, did not demonstrate significant therapeutic benefit in the same ALS murine model at a dietary concentration of 25 mg/kg body weight. RSV treatment did not improve survival, neuromuscular performance, or weight maintenance, suggesting that its efficacy may be context‐dependent and influenced by disease‐specific molecular targets [99].

Nonetheless, RSV showed significant benefit in a different model of neuromuscular degeneration. In mdx mice, a model of Duchenne muscular dystrophy, RSV administered at 4 g/kg body weight improved muscle mass and reduced oxidative damage and fibrosis, although it did not reduce immune cell infiltration or TGF‐β1 expression. These partial effects nonetheless underscore RSV's therapeutic potential in specific pathological contexts [100].

Taken together, these findings support the therapeutic promise of polyphenols, either as isolated compounds or in synergistic formulations, for the treatment of skeletal muscle disorders. Their ability to modulate oxidative stress, inflammation, mitochondrial function, metabolic pathways, and even neuromuscular health forms a compelling rationale for their translation into clinical trials aimed at preventing or treating muscle‐related diseases in humans.

## 5 Human Intervention Studies: Challenges and Future Perspectives

Although there is an extensive literature demonstrating the beneficial effects of polyphenols on various human pathologies, including those affecting skeletal muscle, most of the current evidence stems from in vitro studies or, in vivo research in murine models. Data derived from clinical practice or human trials remain relatively scarce and fragmented (Table 1). Moreover, substantial heterogeneity exists across studies regarding polyphenol sources, dosages, formulations, treatment durations, and study designs, as well as in participant characteristics such as age, sex, baseline health status, and level of physical activity. Outcome measures also vary widely, ranging from molecular biomarkers to functional performance assessments, which complicates direct comparisons across studies. Collectively, these factors limit the immediate translational applicability of preclinical findings and highlight the need for rigorously designed, adequately powered, and standardized clinical trials to establish clear evidence of efficacy in humans.

**TABLE 1:** Overview of the references reported in the manuscript and details on clinical trials and human interventions.

Study type	Number of references	Examples of references
in vitro (cell culture)	35	**[43]; [44]; [54‐66]; [69]; [70‐78]; [81‐88]; [90]; [101]**
in vivo (animal studies)	22	**[41–45]; [67–73]; [75]; [85]; [87–90]; [97–100]**
clinical / human trials	6	**[101–106]**

*Details on clinical trials and human interventions*:							
Ref.	Authors	Year	Population	Intervention	Control	Outcomes	Results
**101**	Tanaka et al.	2024	healthy adults	piceatannol	Placebo	*SIRT1* expression	↑ *SIRT1* levels
**102**	Alway et al.	2017	older men and women	resveratrol and physical exercise	placebo/exercise	muscle functions	↑ adaptations
**103**	Sgrò et al.	2021	young men	quercetin	placebo	IGF‐I/II CK, LDH, IL6	↑ IGFI/II ↓ CK, LDH, IL6
**104**	Bazzucchi et al.	2020	healthy men	quercetin	placebo	NM* recovery CK, LDH	↑ performances ↓ CK, LDH
**105**	Bazzucchi et al.	2019	healthy men	quercetin	placebo	NM* damage	↓ damage
**106**	Patrizio et al.	2018	healthy men	quercetin	placebo	muscle performances	↑ performances

*Note*: only original research articles mentioned in the manuscript are included in the table

Recognizing these limitations is essential to contextualize the current evidence base, guide future research priorities, and provide a balanced understanding of the potential of polyphenols in supporting human skeletal muscle health and athletic performance. In a recent study, Tanaka and colleagues [101], investigated the effects of piceatannol, a polyphenol abundant in passion fruit seeds, not only in in vitro, but also in a small human cohort. Subjects consumed a test food containing 100 mg of piceatannol daily for 2 weeks. The results revealed a significant upregulation of SIRT1 expression in whole blood among the treated group compared to controls, suggesting that piceatannol may exert bioactive effects in humans. Similarly, Alway et al. [102] explored the impact of RSV supplementation in older adults aged 65–80 years undergoing a 12‐week physical training program. Participants were divided into two groups: one receiving RSV at 500 mg/day in addiction to exercise, and the other undergoing exercise alone. The combination of RSV physical training led to enhanced mitochondrial density, improved resistance to fatigue, greater myofiber cross‐sectional area, and increase myonuclei count, compared to training alone.

Furthermore, while training alone did not affect peak torque and power output of the knee extensor muscle, RSV co‐treatment increased these parameters by 8%, and 14%, respectively, highlighting its potential synergistic role in enhancing muscle function. Quercetin, another well studied polyphenol, has also demonstrated promising outcome in human studies. In one study, 12 young adults supplemented with 1 gram per day of quercetin for 14 days prior to eccentric exercise‐induced muscle damage, exhibited reduced markers of muscle injury, such as creatine kinase (CK), lactate dehydrogenase (LDH), and myoglobin (Mb) as well as lower levels of IL‐6 and improved modulation of IGF‐I and IGF‐II, all of which contributed to better physical performance [103].

Additional studies further confirmed the role of quercetin in improving neuromuscular recovery [104], and attenuating muscle weakness following intense exercise [105]. In a randomized, double‐blind, crossover trial involving 10 healthy men, a single 1‐gram dose of quercetin taken 3 h before resistance training significantly enhanced neuromuscular performance, reduced muscle soreness, and lowered CK levels compared to placebo [106].

Despite these encouraging findings, several major challenges limit the widespread clinical use of polyphenol‐based therapies. These include low bioavailability, instability, extensive first‐pass metabolism, and inter‐individual variability in response. Most polyphenols are poorly water‐soluble and thus exhibit limited intestinal absorption. Moreover, their structural integrity is often compromised by pH fluctuations, light exposure, or thermal conditions, factors that can negatively affect their biological activity. Metabolic transformations by the gut microbiota and hepatic enzymes can further alter polyphenol structures, complicating pharmacokinetic tracking and efficacy assessment [107].

An important point that must be emphasized is that in many in vitro studies on cells or in vivo studies on animal models, the amount of polyphenols administered, either individually or in combination, often exceeds what can be achieved through a normal diet or specific supplementation, making these studies not always directly translatable to humans [108, 109].

Although the concentrations used in vitro are often high, these experiments allow the identification of molecular pathways and potential biological targets of polyphenols that may be relevant even at lower in vivo concentrations, considering, for example, tissue accumulation and the activity of active metabolites. Indeed, the absorption and distribution of polyphenols in target tissues can differ significantly from plasma concentrations, supporting the notion that plasma levels do not fully reflect the actual exposure to the compounds or their metabolites in the tissues directly involved in biological processes [110].

Several studies have shown that certain polyphenols are transformed through interactions with other molecules [111] or by the action of phase I and II enzymes, while still retaining biological activities similar to, or even greater than, those of the parent compounds [112].

The action of the gut microbiota also plays a crucial role in these processes. Some polyphenols that reach the colon can be metabolized by intestinal microorganisms into low‐molecular‐weight compounds, which have an enhanced ability to cross biological barriers and may achieve systemic and tissue concentrations different from their respective precursors. Indeed, after ingestion, not only the parent compounds but also their metabolites or conjugated forms appear in plasma and can contribute to the biological effects observed in vivo [113].

A full understanding of these mechanisms is essential for the design and progression of future studies that take into account the complex network of interactions capable of influencing the cellular and molecular mechanisms underlying the activity of individual polyphenols or their mixtures.

Another important yet often overlooked challenge is the practical difficulty of extracting and purifying polyphenols in sufficient quantities and at reasonable costs for clinical application or routine supplementation. To address these limitations, various strategies are currently under investigation. These include the development of eco‐friendly, cost‐effective extraction methods [114], as well as the encapsulation of polyphenols in nanoparticles or other nanocarriers to enhance stability, protect them against degradation, and facilitate targeted cellular delivery [115]. These innovations are expected to significantly improve the efficacy, bioavailability, and clinical applicability of polyphenol‐based interventions. In conclusion, while much remains to be explored, polyphenols hold promising therapeutic potential for the prevention and treatment of skeletal muscle disorders. Advances in formulation science, delivery systems, and clinical research are likely to pave the way for more effective and sustainable strategies to harness these natural compounds in safeguarding muscle health and overall physiological well‐being.

## 6 Conclusions

In conclusion, a growing body of scientific evidence increasingly describes polyphenols as promising modulators of skeletal muscle development and homeostasis, acting through multiple signaling pathways and cellular mechanisms that regulate proliferation, differentiation, and adaptation. Experimental data suggest that these bioactive compounds not only support physiological muscle function but may also represent innovative therapeutic strategies to prevent or mitigate age‐related muscle disorders.

However, fully harnessing the potential of polyphenols for maintaining muscle health in clinical practice still requires further in‐depth studies aimed at defining optimal dosages, bioavailability, and interactions with phase I and II enzymes, other molecules or nutrients, and the gut microbiota.

## Conflicts of Interest

The authors declare no conflicts of interest.
